# Prognostic irrelevance of plaque vulnerability following plaque sealing in high-risk patients with type 2 diabetes: an optical coherence tomography study

**DOI:** 10.1186/s12933-020-01168-4

**Published:** 2020-11-12

**Authors:** Rosalia Dettori, Andrea Milzi, Kathrin Burgmaier, Mohammad Almalla, Martin Hellmich, Nikolaus Marx, Sebastian Reith, Mathias Burgmaier

**Affiliations:** 1 Department of Internal Medicine I, University Hospital of the RWTH Aachen, Pauwelsstr. 30, 52074 Aachen, Germany; 2 Department of Pediatrics, University Hospital of Cologne, Cologne, Germany; 3 Institute of Medical Statistics and Computational Biology, Faculty of Medicine and University Hospital Cologne, University of Cologne, Cologne, Germany

**Keywords:** Optical coherence tomography, Coronary plaque morphology, Type 2 diabetes mellitus, Plaque sealing

## Abstract

**Background:**

Type 2 diabetes mellitus (T2DM) is associated with an increased cardiovascular risk related at least in part to a more vulnerable plaque phenotype. However, patients with T2DM exhibit also an increased risk following percutaneous coronary intervention (PCI). It is unknown if plaque vulnerability of a treated lesion influences cardiovascular outcomes in patients with T2DM. In this study, we aimed to assess the association of plaque morphology as determined by optical coherence tomography (OCT) with cardiovascular outcome following PCI in high-risk patients with T2DM.

**Methods:**

81 patients with T2DM and OCT-guided PCI were recruited. Pre-interventional OCT and systematic follow-up of median 66.0 (IQR = 8.0) months were performed.

**Results:**

During follow-up, 24 patients (29.6%) died. The clinical parameters age (HR 1.16 per year, 95% CI 1.07–1.26, p < 0.001), diabetic polyneuropathy (HR 3.58, 95% CI 1.44–8.93, p = 0.006) and insulin therapy (HR 3.25, 95% CI 1.21–8.70, p = 0.019) predicted mortality in T2DM patients independently. Among OCT parameters only calcium-volume-index (HR 1.71 per 1000°*mm, 95% CI 1.21–2.41, p = 0.002) and lesion length (HR 1.93 per 10 mm, 95% CI 1.02–3.67, p = 0.044) as parameters describing atherosclerosis extent were significant independent predictors of mortality. However, classical features of plaque vulnerability, such as thickness of the fibrous cap, the extent of the necrotic lipid core and the presence of macrophages had no significant predictive value (all p = ns).

**Conclusion:**

Clinical parameters including those describing diabetes severity as well as OCT-parameters characterizing atherosclerotic extent but not classical features of plaque vulnerability predict mortality in T2DM patients following PCI. These data suggest that PCI may provide effective plaque sealing resulting in limited importance of local target lesion vulnerability for future cardiovascular events in high-risk patients with T2DM.

## Background

Type 2 diabetes mellitus (T2DM) is associated with an increased cardiovascular risk [1, 2] and a higher propensity to develop vulnerable plaques [1–4]. Cardiovascular events account for a major part of morbidity and mortality in patients with T2DM, resulting in a two- to threefold higher mortality risk in these patients in comparison to patients without T2DM [4–6].

Acute coronary syndromes (ACS) are acute manifestations of coronary artery disease [7] and can arise from the rupture of vulnerable coronary lesions [8, 9]. These vulnerable plaques are characterized by various features of plaque vulnerability, including a large necrotic lipid core, an overlying thin fibrous cap and enhanced plaque macrophage infiltration [8]. Optical coherence tomography (OCT) is a novel intravascular imaging modality with supreme resolution which allows visualization and quantification of those vulnerable plaque characteristics [4]. Recently, OCT has been employed in multiple studies to identify features of plaque vulnerability [3, 4, 8, 10–18]. An alternative to OCT is 320-row coronary CT, a non-invasive imaging technique which allows to detect certain features of plaque vulnerability with good accuracy [19].

Previously, Stone et al. [20] employed intravascular ultrasonographic imaging to identify plaque-morphological risk factors for future cardiovascular events. Remarkably, coronary lesions which caused future events were characterized by a small luminal area, a large plaque burden and a thin-cap fibroatheroma with a thin fibrous cap overlying a necrotic lipid core [20]. Calcification morphology, in particular small calcification particles (micro- or spotty calcifications), has also been related to plaque vulnerability [11, 12, 17, 21]. Interestingly, vulnerable plaques tend to cluster in specific sites of coronary arteries and to be more prevalent in segments with tight stenoses [22]. Recently, the LPF-study using intravascular ultrasonography and near-infrared spectroscopy as well as the CLIMA-study employing OCT demonstrated, that the presence of features of coronary plaque vulnerability is associated to a higher incidence of major cardiovascular events [13, 14]. Patients with type 2 diabetes mellitus (T2DM) have a high tendency for presence of coronary vulnerable plaques, which attributes at least in part to the high cardiovascular risk in these patients. For example, previous studies could show that coronary lesions of T2DM patients are characterized by a thinner minimal fibrous cap with a larger lipid core [4, 16]. In addition, occurrence of macrophages and/or of microvessels in T2DM patients indicate the presence of a coronary inflammatory process [4, 16]. Further studies revealed that duration of T2DM is associated with increased plaque vulnerability [23]. The impact of glycemic dysregulation on plaque vulnerability already starts with pre-diabetes [24]. Of course, other risk factors, such as hypercholesterinemia, also influence plaque phenotype—for instance, a study by Yonetsu et al. [25] could show that patients with low LDL-cholesterol more often present a plaque erosion rather than plaque rupture as the morphologic correlate of acute coronary syndromes.

Whereas these vulnerable plaques are prone to plaque rupture with consecutive acute cardiovascular events in the natural history course of atherosclerosis, the clinical consequence to seal these vulnerable plaques using percutaneous coronary intervention (PCI) and stent implantation is unclear. Therefore, in this study we aimed to analyze both pre-PCI plaque morphology, assessed using OCT, as well as clinical characteristics as predictors for mortality in high-risk T2DM patients following PCI.

## Methods

### Study population

We enrolled 81 patients with known T2DM undergoing coronary angiography with OCT-guided PCI at the Department of Internal Medicine I, University Hospital of the RWTH Aachen, Germany between May 2011 and January 2014. Indication for coronary angiography and PCI was ACS in 27 patients and stable angina in 54 patients. Stable coronary artery disease (CAD) was defined as no progression of symptoms in the last 6 weeks; in this case, identification of the target lesion relied on stress imaging and/or FFR. Previous studies partially relied on these data [3, 4, 9, 12, 17, 18]. Further inclusion criteria were age > 30 years and written informed consent to the study protocol. Exclusion criteria included left main coronary artery stenosis, graft stenosis, hemodynamic or rhythmic instability, acute or chronic renal insufficiency (serum creatinine level > 1.5 mg/dl), systemic acute or chronic infections, pregnancy, anti-inflammatory medications such as steroids and chronic total occluded, severely tortuous or calcified vessels, which impeded proper advancement of the OCT catheter. A further exclusion criterion was incomplete follow-up data; these were acquired telephonically using a standardized questionnaire recording, among other, death of the patient and time of death. Furthermore, cardiovascular death was recorded; this was defined as death resulting from myocardial infarction, sudden cardiac death, death due to heart failure, death due to stroke, death due to cardiovascular procedures, death due to cardiovascular hemorrhage, and death due to other cardiovascular causes, according to previous definitions [26].

The study was approved by the local Ethics Committee and conforms with the Declaration of Helsinki on ethical principles for medical research involving human subjects.

### OCT image acquisition and analysis

OCT image acquisition of the target vessel was performed by using the Frequency Domain-OCT C7XR system and the DragonFly catheter (St. Jude Medical, LightLab Imaging Inc, Westford, Massachusetts, USA) as previously described [27]. Target/culprit lesion was defined as previously described [4]. OCT-image analysis was executed throughout the entire lesion frame by frame in a 0.2 mm interval by two independent observers applying the proprietary software provided by LightLab Imaging. Determination of intraluminal and plaque morphologic parameters by OCT occurred as previously described [4].

### Statistical analysis

Statistical analysis was performed using SPSS Statistics (IBM Corp., Armonk, NY, USA). Categorical variables are reported as n and percentage, continuous variables as mean ± standard deviation. A comparison of continuous variables was performed with t-test, while a comparison of nominal variables took place using Pearson’s χ-squared test.

Follow-up duration was determined by reverse Kaplan–Meier analysis. Uni- and multivariable Cox regression analyses were performed to calculate hazard ratios (HR) for clinical and OCT-derived parameters to predict mortality. For multivariable analysis, significant predictors in the univariable analysis were included in the model and then eliminated stepwise through backward-selection. In order to avoid collinearity of two or more parameters, a selection according to statistical and biological significance was performed before inclusion in the multivariable model. Specifically, we selected calcium volume index among all parameters assessing calcium quantity in the target/culprit lesion which were significant in the univariable analysis (presence of a calcific plaque, presence of macrocalcifications, number of macrocalcifications, mean calcium arc, calcium length). In summary, we included several clinical (age, BMI, HDL-c, GFR, diabetic polyneuropathy, insulin therapy, metformin therapy) and plaque-morphological (lesion length, stenosis eccentricity, calcium volume index) parameters in the multivariable Cox regression model. These parameters were then selected stepwise backwards if presenting p > 0.10; this resulted in a multivariable model including 5 variables (age, diabetic polyneuropathy, insulin therapy, lesion length, calcium volume index). The final model was validated, accounting for the variable selection process, by the bootstrap as proposed by Harrell [28]. Beforehand, missing values were multiply imputed (5 sets). Optimism in the c-statistic (area under ROC curve) was about 6%, e.g. from 0.86 (original) to 0.81 (bias-corrected) with a slope of 0.61. Thus, model coefficients should be shrinked by 39% (multiply by 0.61) to get better predictions. For plaque morphological features relevantly associated to excess mortality, we performed receiver operating curve (ROC) analysis and determined optimal cut-off values for mortality after 5 years. Optimal cut-off was defined as the value with the highest Youden index; diagnostic efficiency was classified as previously described [29]. Kaplan–Meier analyses were performed using these cut-off values. Statistical significance was awarded by a p-value < 0.05.

## Results

### Baseline characteristics

**Fig. 1:**
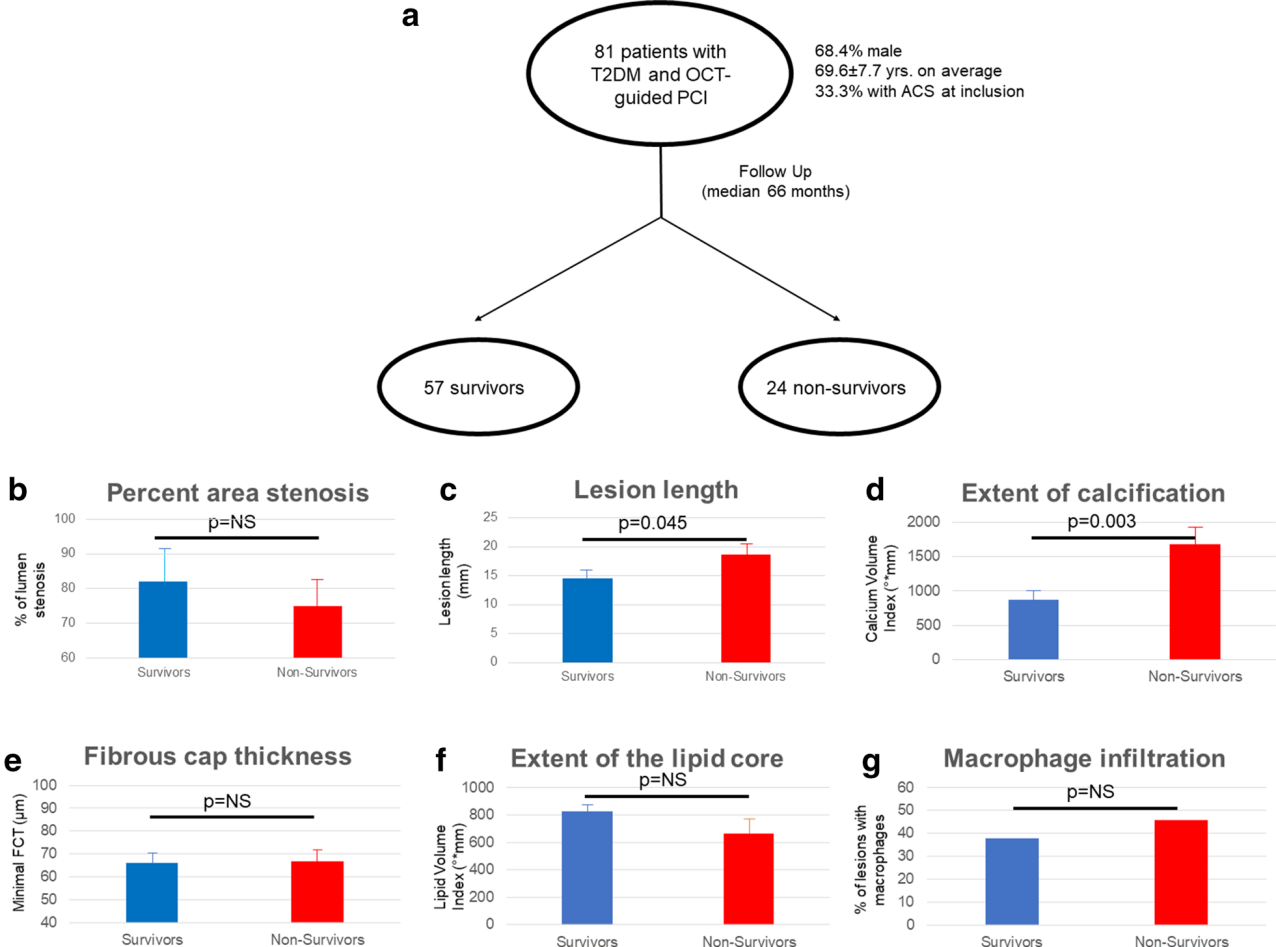
Study population and plaque-morphologic characteristics of survivors vs. non-survivors. In **a**, details of inclusion are shown. Graphical representation of differences in plaque composition are shown in **b**–**g** as means ± SEM or percentage; survivors are depicted in blue, non-survivors in red

A total of 81 patients with T2DM underwent coronary angiography due to stable/unstable CAD. Following OCT-guided PCI, patients were followed for a median follow-up of 66.0 (IQR = 8.0) months. In this period of time, 24 (29.6%) patients died. The baseline patient and lesion characteristics are shown in Tables 1 and 2, including a comparison of non-survivors with survivors. The most relevant data are also graphically shown in Fig. 1.

**Table 1:** Patient characteristics and comparison between survivors and non-survivors

	Study population	Survivors	Non-survivors	
	n = 81	n = 57	n = 24	p
Age (years)	69.6 ± 7.7	67.8 ± 7.8	74.0 ± 5.6	0.001
Sex (male, n, %)	54 (66.7)	39 (68.4)	16 (66.7)	0.606
ACS at presentation (n, %)	27 (33.3)	19 (33.3)	8 (33.3)	1.000
CV risk profile				
BMI (kg/m^2^)	30.7 ± 5.4	31.4 ± 5.5	28.9 ± 3.9	0.050
Waist circumference (cm)	107.6 ± 13.2	108.2 ± 14.4	106.3 ± 9.8	0.562
Hypertension (n, %)	70 (86.4)	50 (87.7)	20 (83.3)	0.599
Hyperlipidemia (n, %)	58 (71.6)	42 (73.7)	16 (66.7)	0.522
Total cholesterol (mg/dl)	190.0 ± 43.1	190.0 ± 43.1	190.0 ± 43.1	0.407
LDL-c (mg/dl)	117.0 ± 35.1	115.1 ± 34.4	121.7 ± 37.2	0.450
HDL-c (mg/dl)	43.3 ± 10.8	41.9 ± 9.5	46.7 ± 13.0	0.066
Triglycerides (mg/dl)	173.1 ± 75.8	175.9 ± 78.0	166.7 ± 71.6	0.623
Active smoking (n, %)	17 (21.0)	12 (21.1)	5 (20.8)	0.982
Pack years (py)	19.3 ± 21.8	19.5 ± 23.0	18.7 ± 19.1	0.879
Family history of CAD (n, %)	37 (45.7)	31 (54.4)	6 (25.0)	0.015
Previous CAD (n, %)	30 (37.0)	18 (31.6)	12 (50.0)	0.117
Previous PCI (n, %)	24 (29.6)	14 (24.6)	10 (41.7)	0.124
Previous CABG (n, %)	2 (2.5)	1 (1.8)	1 (4.2)	0.523
hsCRP (mg/dl)	13.6 ± 22.5	12.4 ± 23.1	16.6 ± 21.1	0.448
GFR (mg/dl/1.73 m^2^)	58.8 ± 5.2	59.5 ± 3.8	57.1 ± 7.3	0.136
Other comorbidities				
COPD (n, %)	11 (13.6)	7 (12.3)	4 (16.7)	0.599
Diabetes severity and therapy				
Diabetes duration (years)	11.1 ± 10.0	10.3 ± 9.9	13.1 ± 9.9	0.250
Diabetic polyneuropathy (n, %)	23 (28.4)	12 (21.1)	11 (45.8)	0.024
Diabetic retinopathy (n, %)	14 (17.3)	8 (14.0)	6 (25.0)	0.233
HbA1c (%)	7.1 ± 1.0	7.0 ± 1.0	7.3 ± 1.0	0.155
Insulin (n,%)	34 (42.0)	18 (31.6)	16 (66.7)	0.003
Metformin (n,%)	52 (64.2)	40 (71.4)	12 (50.0)	0.066
Sulfonylamides (n, %)	17 (21.0)	13 (22.8)	4 (16.7)	0.535
Incretins (n, %)	15 (18.5)	8 (14.0)	7 (29.2)	0.109
Other relevant medication				
Aspirin (n, %)	77 (95.1)	54 (94.7)	23 (95.8)	0.835
Statin (n, %)	54 (66.6)	40 (71.4)	14 (58.4)	0.137
ACE or ARBi (n, %)	58 (71.6)	42 (76.4)	16 (66.7)	0.370
Beta-blocker (n, %)	62 (76.5)	46 (80.7)	16 (66.7)	0.173

*ACS* acute coronary syndrome, *BMI* body mass index, *CAD* coronary artery disease, *PCI* percutaneous coronary intervention, *CABG* coronary artery bypass graft, *GFR* glomerular filtration rate, *COPD* chronic obstructive pulmonary disease, *ACE* angiotensin converting enzyme, *ARB* angiotensin receptor blocker

**Table 2:** Lesion characteristics and comparison between survivors and non-survivors

	Study population	Survivors	Non-survivors	p
	n = 81	n = 57	n = 24	
Vessel				
LAD (n, %)	35 (43.2)	24 (42.2)	11 (45.8)	0.558
CX (n, %)	13 (14.8)	11 (19.3)	1 (4.2)	0.558
RCA (n, %)	30 (37.0)	19 (33.3)	11 (45.8)	0.558
RIM (n, %)	4 (4.9)	3 (5.3)	1 (4.2)	0.558
Stenosis characteristics				
Percent area stenosis (%)	76.8 ± 8.0	82.0 ± 10.9	75.0 ± 15.6	0.536
MLA (mm^2^)	1.4 ± 0.6	1.4 ± 0.7	1.3 ± 0.5	0.704
MLD (mm)	1.1 ± 0.3	1.1 ± 0.3	1.0 ± 0.2	0.319
Stenose lumen eccentricity (%)	25.4 ± 11.9	23.4 ± 10.2	30.2 ± 14.5	0.051
Lesion morphology				
Lesion length (mm)	15.7 ± 8.4	14.5 ± 8.1	18.7 ± 8.5	0.045
Lipid plaque (n,%)	44 (54.3)	33 (57.9)	11 (47.8)	0.413
Calcific plaque (n,%)	52 (64.2)	32 (56.1)	20 (87.0)	0.009
Fibrous plaque (n,%)	71 (87.7)	50 (87.7)	21 (91.3)	0.646
Presence of TCFA (n, %)	30 (37.0)	23 (40.4)	7 (33.3)	0.823
Minimal FCT (µm)	66.3 ± 26.5	66.2 ± 26.1	66.8 ± 28.8	0.945
Mean FCT (µm)	111.3 ± 29.3	108.4 ± 25.9	120.3 ± 38.1	0.244
Lipid arc (°)	147.6 ± 45.8	151.3 ± 45.8	136.1 ± 46.0	0.343
Lipid volume index (°*mm)	787.2 ± 477.0	824.9 ± 481.8	666.9 ± 462.1	0.344
Presence of microchannels (n, %)	37 (45.7)	26 (46.4)	11 (47.8)	0.910
Calcification morphology				
Presence of microcalcifications (n, %)	15 (18.5)	11 (21.6)	4 (16.7)	0.621
Number of microcalcifications	0.3 ± 0.7	0.3 ± 0.7	0.3 ± 0.7	0.902
Presence of spotty calcifications (n, %)	60 (74.1)	43 (84.3)	17 (70.8)	0.173
Number of spotty calcifications	2.1 ± 2.0	2.2 ± 2.1	1.8 ± 1.6	0.388
Presence of macrocalcifications (n,%)	58 (71.6)	35 (68.6)	23 (95.8)	0.009
Number of macrocalcifications	1.5 ± 1.3	1.2 ± 1.0	2.3 ± 1.5	< 0.001
Total number of calcifications	4.0 ± 2.7	3.7 ± 2.8	4.4 ± 2.4	0.310
Mean calcium arc (°)	79.4 ± 39.8	71.6 ± 33.7	95.9 ± 46.8	0.013
Calcium length (mm)	12.3 ± 8.3	10.7 ± 8.0	15.9 ± 8.3	0.010
Calcium volume index (°*mm)	1133.5 ± 1093.1	877.4 ± 908.5	1677.9 ± 1263.2	0.003
Plaque inflammation				
Presence of macrophages (n, %)	31 (38.3)	20 (37.7)	11 (45.8)	0.502
Macrophage arc (°)	15.1 ± 21.7	15.7 ± 23.4	13.7 ± 17.7	0.704
Macrophage length (mm)	0.8 ± 1.2	0.8 ± 1.3	0.6 ± 0.8	0.358
Macrophage volume index (°*mm)	39.5 ± 15.7	41.0 ± 77.8	20.5 ± 32.0	0.109

*LAD* left anterior descending, *CX* ramus circumflexus, *RCA* right coronary artery, *RIM* ramus intermedius, *MLA* minimal luminal area, *MLD* minimal luminal diameter, *TCFA* thin capped fibroatheroma, *FCT* fibrous cap thickness

### Clinical and morphologic predictors of mortality following PCI

In order to find predictors of mortality following PCI, univariable Cox regression analyses were performed. We found several clinical (Table 3) and morphological plaque characteristics (Table 4) to be predictors of mortality following PCI. However, classical features of plaque vulnerability such as thickness of the fibrous cap overlying the necrotic lipid core, the extent of the necrotic lipid core, the presence of micro- or spotty calcifications and the presence of macrophages had no significant predictive value for mortality (all p = ns), neither in patients with ACS nor in patients with stable CAD (p = ns).

**Table 3:** Univariable Cox-regression analysis for clinical parameters as predictors of mortality following coronary intervention

	HR (95% CI)	p
Age (per year)	1.11 (1.04–1.18)	0.001
Sex (male)	0.65 (0.28–1.51)	0.320
ACS at presentation (presence)	0.99 (0.41–2.41)	0.983
CV risk profile		
BMI (per kg/m^2^)	0.91 (0.82–1.00)	0.059
Waist circumference (per 10 cm)	0.94 (0.68–1.30)	0.718
Hypertension (presence)	0.94 (0.28–3.17)	0.923
Hyperlipidemia (presence)	0.70 (0.30–1.66)	0.704
Total cholesterol (per 10 mg/dl)	1.05 (0.95–1.16)	0.313
LDL-c (per 10 mg/dl)	1.07 (0.94–1.21)	0.311
HDL-c (per 10 mg/dl)	1.43 (1.02–1.99)	0.036
Triglycerides (per 10 mg/dl)	0.98 (0.92–1.04)	0.447
Active smoking (presence)	0.99 (0.37–2.66)	0.981
Pack years (per 10 py)	0.95 (0.79–1.16)	0.637
Family history of CAD (presence)	0.35 (0.14–0.89)	0.351
Previous CAD (presence)	1.99 (0.88–4.52)	0.099
Previous PCI (presence)	1.93 (0.85–4.41)	0.118
Previous CABG (presence)	2.29 (0.31–17.1)	0.418
hsCRP (per 10 mg/dl)	1.06 (0.93–1.22)	0.375
GFR (per 10 mg/dl/1.73 m^2^)	0.49 (0.27–0.90)	0.020
Other comorbidities		
COPD (presence)	1.30 (0.44–3.48)	0.638
Diabetes severity and therapy		
Diabetes duration (per year)	1.03 (0.99–1.06)	0.125
Diabetic polyneuropathy (presence)	2.94 (1.29–6.68)	0.010
Diabetic retinopathy (presence)	1.90 (0.75–4.82)	0.178
HbA1c (per 1%)	1.34 (0.92–1.97)	0.131
Insulin (presence)	3.44 (1.45–8.13)	0.005
Metformin (presence)	0.40 (0.17–0.90)	0.028
Sulfonylamides (presence)	0.73 (0.25–2.16)	0.575
Incretins (presence)	2.11 (0.87–5.14)	0.100
Other relevant medication		
Aspirin (presence)	1.21 (0.16–8.96)	0.854
Statin (presence)	0.54 (0.24–1.24)	0.149
ACE or ARBi (presence)	0.76 (0.31–1.85)	0.547
Beta-blocker (presence)	0.52 (0.22–1.24)	0.142

*ACS* acute coronary syndrome, *BMI* body mass index, *CAD* coronary artery disease, *PCI* percutaneous coronary intervention, *CABG* coronary artery bypass graft, *GFR* glomerular filtration rate, *COPD* chronic obstructive pulmonary disease, *ACE* angiotensin converting enzyme, *ARB* angiotensin receptor blocker

**Table 4:** Univariable Cox-regression analysis for plaque-morphological parameters as predictors of mortality following coronary intervention

	HR (95% CI)	p
Stenosis characteristics		
Percent area stenosis (per 10%)	0.89 (0.54–1.48)	0.665
MLA (per mm^2^)	0.88 (0.45–1.73)	0.711
MLD (per mm)	0.31 (0.04–2.41)	0.264
Stenosis lumen eccentricity (per 10%)	1.61 (1.14–2.28)	0.007
Lesion morphology		
Lesion length (per 10 mm)	1.69 (1.07–2.68)	0.026
Lipid plaque (presence)	0.78 (0.34–1.80)	0.559
Calcific plaque (presence)	4.06 (1.20–13.72)	0.024
Fibrous plaque (presence)	1.29 (0.30–5.52)	0.733
Presence of TCFA (presence)	0.87 (0.32–2.34)	0.871
Minimal FCT (per 10 µm)	0.98 (0.78–1.23)	0.877
Mean FCT (per 10 µm)	1.10 (0.91–1.33)	0.336
Lipid arc (per 10°)	0.95 (0.83–1.08)	0.435
Lipid volume index (per 100°*mm)	0.95 (0.84–1.09)	0.481
Presence of microchannels (presence)	0.99 (0.43–2.28)	0.974
Calcification morphology		
Presence of microcalcifications (presence)	0.71 (0.24–2.10)	0.539
Number of microcalcifications (per calcification)	0.93 (0.51–1.69)	0.932
Presence of spotty calcifications (presence)	0.52 (0.22–1.28)	0.155
Number of spotty calcifications (per calcification)	0.90 (0.71–1.14)	0.386
Presence of macrocalcifications (presence)	8.23 (1.11–61.11)	0.039
Number of macrocalcifications (per calcification)	1.43 (1.14–1.79)	0.002
Total number of calcifications (per calcification)	1.06 (0.92–1.22)	0.433
Mean calcium arc (per 10°)	1.10 (1.03–1.19)	0.005
Calcium length (per mm)	1.06 (1.02–1.11)	0.008
Calcium volume index (per 1000°*mm)	1.62 (1.21–2.17)	0.001
Plaque inflammation		
Presence of macrophages (presence)	1.22 (0.54–2.77)	0.632
Macrophage arc (per 10°)	0.95 (0.78–1.15)	0.605
Macrophage length (per mm)	0.84 (0.38–1.24)	0.385
Macrophage volume index (per 100°*mm)	0.59 (0.24–1.42)	0.233

*MLA* minimal luminal area, *MLD* minimal luminal diameter, *TCFA* thin capped fibroatheroma, *FCT* fibrous cap thickness

To evaluate which of the above-mentioned parameters independently predict mortality following PCI, multivariable analysis was performed. In this analysis the clinical parameters age (HR 1.16 per year, 95% CI 1.07–1.26, p < 0.001), diabetic polyneuropathy (HR 3.58, 95% CI 1.44–8.93, p = 0.006) and insulin therapy (HR 3.25, 95% CI 1.44–8.70, p = 0.019) were significant predictors of mortality. Regarding plaque characteristics, only calcium volume index (HR 1.71 per 1000°*mm, 95% CI 1.21–2.41, p = 0.002) and lesion length (HR 1.93 per 10 mm, 95% CI 1.02–3.67, p = 0.044) were independent significant morphological predictors of mortality following PCI.

To further analyze the role of these parameters in predicting cardiovascular events, we focused on the prediction of cardiovascular mortality. Here, only age (HR 1.14 per year, 95% CI 1.02–1.28, p = 0.018) and calcium volume index (HR 2.04 per 1000°*mm, 95% CI 1.29–3.23, p = 0.002) could predict cardiovascular mortality in univariable Cox regression analysis. Performing multivariable Cox regression analysis, both age (HR 1.16 per year, 95% CI 1.02–1.32, p = 0.025) and calcium volume index (HR 1.94 per 1000°*mm, 95% CI 1.24–3.02, p = 0.003) still remained significant independent predictors of cardiovascular mortality.

### Diagnostic value of morphologic predictors of mortality following PCI

**Fig. 2:**
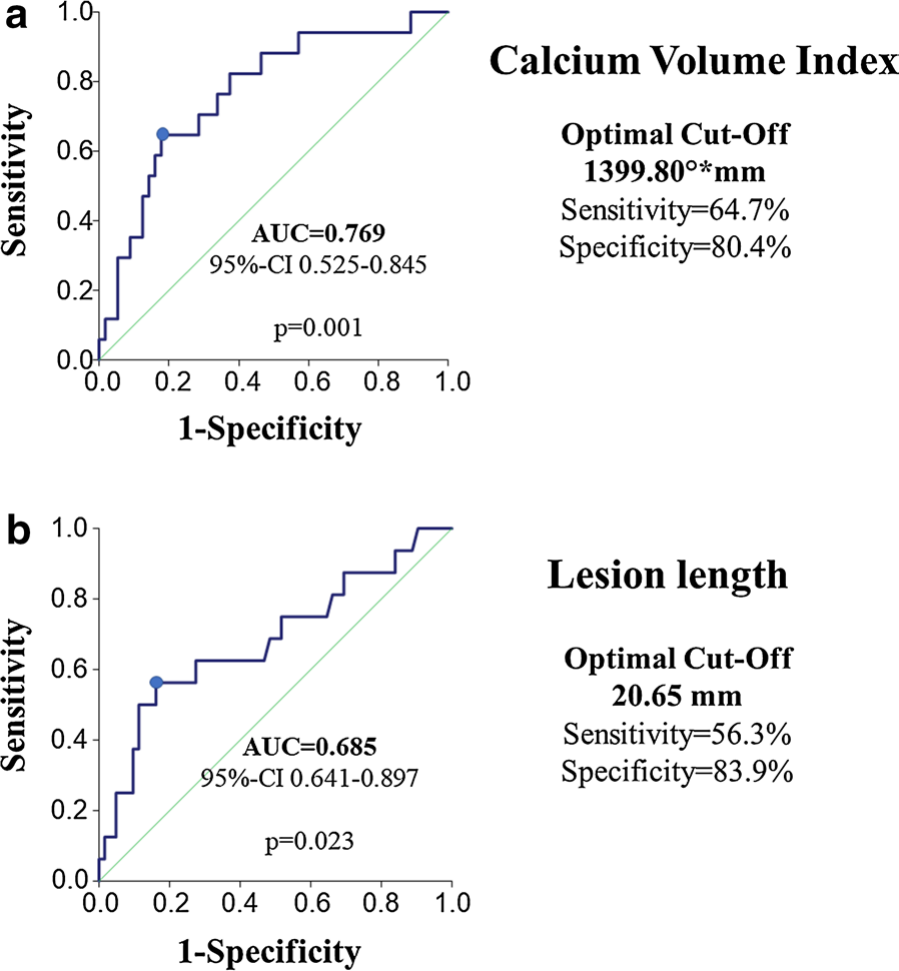
ROC-curves for calcium volume index (**a**) and lesion length (**b**) as predictors of mortality following PCI. *CVI* calcium volume index

In order to determine the diagnostic value of morphologic predictors of mortality after 5 years, ROC analyses were performed. In these analyses, calcium volume index predicted mortality following PCI with good diagnostic efficiency (AUC 0.769, 95% CI 0.525–0.845, p = 0.001; sensitivity: 64.7%, specificity: 80.4% at the optimal cut-off = 1399.8°*mm). Furthermore, lesion length predicted mortality following PCI with sufficient efficiency (AUC 0.685, 95% CI 0.641–0.897, p = 0.023; sensitivity: 56.3%, specificity: 83.9% at the optimal cut-off = 20.65 mm). The ROC-curves are represented in Fig. 2.

**Fig. 3:**
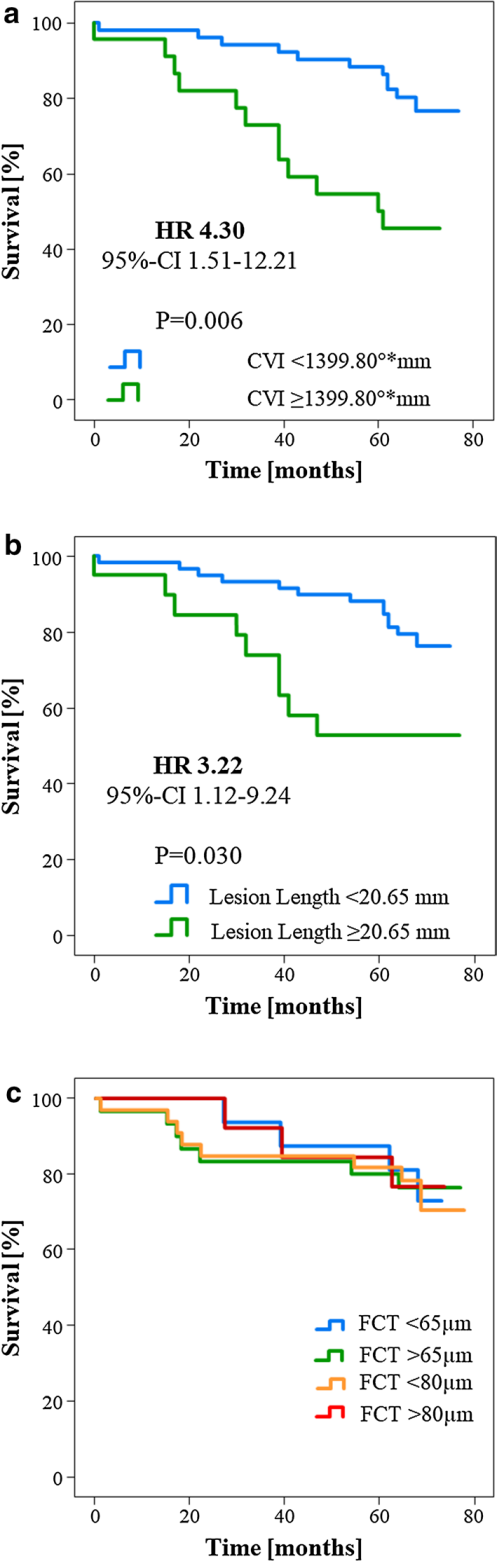
Kaplan–Meier curves for predictors of mortality following PCI. Calcium volume index (**a**) and lesion length (**b**) significantly predict mortality following coronary intervention. On the other hand, minimal FCT (**c**) shows no predictive value following plaque sealing through PCI. *CVI* calcium volume index, *FCT* fibrous cap thickness

To analyze the diagnostic value of these cut-offs to distinguish between high- and low-risk patients, Kaplan–Meier analyses were performed. In these analyses, calcium volume index > 1399.8°*mm and lesion length > 20.65 mm were associated with a hazard ratio of 4.30 (95% CI 1.51–12.21, p = 0.006) and 3.22 (95% CI 1.12–9.24, p = 0.030), respectively. Graphical representation is shown in Fig. 3a, b. In order to assess the diagnostic value of minimal FCT, the most widely accepted parameter of plaque vulnerability, to distinguish between high- and low-risk patients following PCI, we performed Kaplan–Meier analyses using the accepted thresholds of 65 and 80 µm [22]. This showed no significant association of this feature of plaque vulnerability with mortality following PCI (HR 1.09, 95% CI 0.32–3.77, p = 0.885 for FCT < 65 µm; HR 1.20, 95% CI 0.32–4.56, p = 0.787 for FCT < 80 µm, see Fig. 3c).

## Discussion

The main finding of this study is that clinical parameters describing diabetes severity (e.g. diabetic polyneuropathy and insulin therapy) as well as OCT-parameters characterizing atherosclerotic extent (such as lesion length and calcium volume index) but not classical features of plaque vulnerability (e.g. FCT, extent of the necrotic lipid core, presence of macrophages or microcalcifications) predict mortality in T2DM patients following PCI (Fig. 3).

It is well known that cardiovascular morbidity and mortality are significantly enhanced in patients with T2DM [30, 31]. In addition, this increased risk of patients with T2DM persists also following PCI of the target/culprit lesion. As it is unknown if plaque vulnerability of a treated lesion influences cardiovascular outcomes in patients with T2DM, the present study aimed to assess the association of plaque morphology as determined by OCT with cardiovascular outcome following PCI.

In this study, analysis of plaque morphology was performed using OCT, an invasive coronary plaque imaging modality with a supreme resolution, which permits detailed delineation and quantification of intramural dimensions and structures [27, 32, 33]. In our study, we routinely performed pre- and post-interventional OCT. In case of suboptimal PCI-results (stent underexpansion, stent malapposition, relevant stent-edge dissections), PCI optimization was performed on the interventionalist’s discretion. Thus, an effect of suboptimal PCI on the long-term prognosis of our study population can be excluded.

### Advanced T2DM is associated with increased mortality following PCI

In the present study, significant independent clinical predictors of mortality were age, insulin therapy and diabetic polyneuropathy. Whereas age appears quite self-explanatory, the other two factors do require a brief discussion. Our results show that an insulin-based therapy is associated with an increased mortality risk in our patient population. This association is in line with the findings of previous studies in CAD patients [34, 35] and may reflect advanced T2DM requiring insulin therapy. This hypothesis is further supported by the significantly higher mortality risk of patients with diabetic polyneuropathy, as a typical late-onset complication of advanced T2DM. These results are in line with findings of previous studies describing an association of cardiovascular autonomic neuropathy and an excess mortality in patients with T2DM [36–38].

### Advanced atherosclerosis, but not classical parameters of plaque vulnerability predict mortality following PCI

Among all plaque-morphological parameters investigated, only parameters assessing extent of calcification (e.g. calcium volume index) and lesion length as markers of advanced atherosclerosis predicted mortality independently in T2DM patients following PCI.

The extent of coronary calcification has been related to adverse prognosis both in patients with [39] and without known CAD [40]; these studies relied on computed tomography to evaluate the calcium burden in the coronary arteries [39, 40]. However, it is unknown if coronary plaque calcification represents a vulnerable plaque feature in itself or merely reflects more advanced CAD. In addition, semi-quantitative assessment of coronary calcification performed with coronary angiography has been associated with increased mortality following PCI [41]. Our findings are in line with this body of evidence, confirming the negative prognostic role of extensive coronary calcification as determined by OCT. This confirms the hypothesis that a more extensive calcification, which is typical for an advanced stage of atherosclerosis, characterizes a high-risk population.

The length of the atherosclerotic lesion was a further parameter independently associated with mortality in our population. Previous data on this feature are sparce, but in the light of the global findings of our study it is tempting to speculate that lesion length may represent yet another marker of a more diffuse and advanced atherosclerosis as it has been described previously in patients with T2DM [42]. These patients with an advanced atherosclerosis are therefore more frequently prone to further cardiovascular events.

Interestingly, in our study, “classical” features of plaque vulnerability (as FCT, extent of the necrotic lipid core, presence of TCFA, macrophage infiltration or microcalcifications) are not associated with a worse prognosis following PCI of the target/culprit lesion. Previously, Niccoli et al. described in patients with ACS an adverse effect of ruptured plaques compared to plaque erosions on prognosis following primary PCI, which is mainly driven by unstable angina and target vessel failure [43]. As the authors of this study discuss, the different outcomes of patients presenting with plaque rupture and plaque erosion as the morphologic correlate of ACS may be due to a multiplicity of factors, including residual thrombus and/or plaque burden causing suboptimal PCI results through stent malapposition or residual disease at stents’ edge. In the present study, we performed routine PCI optimization, which excluded relevant malapposition or residual plaque burden following intervention. Furthermore, the prognostic relevance of plaque rupture and plaque erosion could not be conclusively determined in our study possibly due to low case number in our mixed cohort of patients with ACS and stable angina. However, in the study by Niccoli et al. no other plaque morphological features were associated with adverse outcome in univariate analysis following PCI, which is in line with the findings of the present study.

Furthermore, the prognostic irrelevance of features of plaque vulnerability following PCI, which we could demonstrate in the present study, may suggest that sealing vulnerable plaques neutralizes its effects on prognosis. The principle of plaque sealing is based on treating plaques by PCI to avoid total vessel occlusion and prevent future events due to plaque rupture or thrombosis [44, 45]. So far, prospective interventional data assessing plaque sealing as a potential strategy to prevent major cardiovascular events in patients with chronic coronary syndrome showed no benefit of such an approach [44]. Remarkably, the lesions treated in this previous study were selected without the use of intravascular imaging and based only on angiographic evidence of intermediate lumen stenosis [44]. This evidence, however, needs to be re-evaluated in the light of the current data. In fact, the CLIMA and LPS studies, in analogy to the data from the PROSPECT study, showed a higher incidence of major cardiovascular events in vulnerable plaques [13, 14, 20]. In contrast, the present study suggests that PCI yields a neutralizing effect on the negative prognostic impact of plaque vulnerability in patients with T2DM. In the light of these novel and potentially clinically relevant findings, the present data highlight the need for prospective, randomized studies evaluating the prognostic effect of vulnerable plaque sealing using OCT-guided PCI. This may be of particular interest in populations with an increased cardiovascular risk, such as patients with T2DM.

## Limitations

A few limitations of our analysis need to be assessed. First, although being the first study correlating clinical and plaque-morphological parameters to survival in patients with T2DM and CAD following PCI, the study population is still relatively small and the findings need to be confirmed in larger cohorts. This is particularly true for the association of plaque morphology with cardiovascular mortality, especially for subgroup analysis. As 3-vessel OCT has not been performed due to ethical reasons, it cannot be excluded that vulnerable coronary plaques other than the culprit/target lesion have not been detected and “sealed”. Also due to ethical reasons we did not include patients with acute or chronic kidney failure, who represent a population with a peculiar risk profile and distinct lesion morphology; therefore, we cannot draw any conclusion regarding these patients. Although we did not detect any association of CRP levels, as the most widely employed inflammation marker, with prognosis following PCI, we do not have data regarding further inflammatory molecules—this will be assessed in further studies.

## Conclusion

Clinical parameters, including those describing diabetes severity, as well as OCT-parameters characterizing atherosclerotic extent but not classical features of plaque vulnerability predict mortality in T2DM patients following PCI. These data suggest that PCI may provide effective plaque sealing, thus potentially counterbalancing the unfavorable effect of local target lesion vulnerability for future cardiovascular events. This finding is of particular importance for patients with T2DM, with the potential to reduce their predisposition to cardiovascular events, which is partly due to a more vulnerable plaque phenotype.

## Data Availability

The datasets used and/or analysed during the current study are available from the corresponding author on reasonable request.

## Abbreviations

ACS: Acute coronary syndrome
CAD: Coronary artery disease
OCT: Optical coherence tomography
PCI: Percutaneous coronary intervention
T2DM: Type 2 diabetes mellitus
